# Effect of Thermal Processing on Flow Properties and Stability of Thickened Fluid Matrices Formulated by Tapioca Starch, Hydroxyl Distarch Phosphate (E-1442), and Xanthan Gum Associating Dysphagia-Friendly Potential

**DOI:** 10.3390/polym13010162

**Published:** 2021-01-04

**Authors:** Huaiwen Yang, Yuhsien Lin

**Affiliations:** Department of Food Science, National Chiayi University, Chiayi City 60004, Taiwan; johnhsien1406@fda.gov.tw

**Keywords:** food thickener, flow behavior, tapioca starch, hydroxypropyl distarch phosphate, xanthan gum, dysphagia

## Abstract

The flow behavior of the administrated fluid matrices demands careful assessments for stability when consumed by individuals with dysphagia. In the present study, we incorporated tapioca starch (TS), hydroxypropyl distarch phosphate (HDP), and xanthan gum (XG) as thickeners into different nectars (300 ± 20 mPa.s) undergoing thermal processing and evaluated their stability. The thickened nectars presented better water holding and oil binding capacities at 25 °C than 4 °C, and the nectars with TS provided the best results for both capacities as well as the highest solubility index and swelling power (*p* < 0.05). All prepared nectars appeared to be shear-thinning fluids with yield stress closely fitting the power law and Casson models. XG-containing nectars presented a higher yield stress and consistency index. Matrices thickened by HDP exhibited a higher viscoelastic property compared to those thickened by TS during thermal processing. TS nectars presented viscous behavior, whereas HDP and XG nectars presented elastic behavior at 80 °C processing. The 3 min thermal processing HDP nectars remained stable and met dysphagia-friendly requirements under 4 °C storage for 28 days, regardless of the type of fluid base (distilled water, sport drink, or orange juice). The employed thickeners present adequate physicochemical properties to be potentially utilized for producing dysphagia-friendly formulations.

## 1. Introduction

Social progress and advances in healthcare and hygiene have promoted a rapid increase in the proportion of elderly individuals worldwide. Consequently, food intake in the elderly has become a topic of considerable interest. Dysphagia, a common condition in the elderly, often causes inadequate dietary intake due to choking, thereby resulting in other problems such as dehydration, malnutrition, weight loss, and aspiration pneumonia [1,2,3].

To resolve the difficulties that elderly individuals face in eating and swallowing, food items are normally softened or thickened by the addition of a liquid. Food thickeners alter the viscosity of liquids, which reduces the flow rate of liquids through the oral cavity or esophagus, promotes the swallowing reflex, and allows ample time for the epiglottis to cover the windpipe, thus reducing the risk of choking [4,5]. In the National Dysphagia Diet guidelines published by the American Dietetic Association, liquid consistencies have been classified into four levels based on viscosity and appearance traits: thin, nectar-like, honey-like, and pudding-like liquids. These levels serve as a reference for the adjustment of diets in care settings [6,7].

Commercial food thickeners required by elderly individuals are mostly available in powder form and can be used to adjust the viscosity of food items by following the manufacturer’s instructions. However, the viscosities obtained after preparation may significantly differ from those stated in the guidelines provided by the manufacturer due to differences in food constituents, preparation temperature, and preparation time, and the prepared products tend to be of unstable quality [8,9,10]. Therefore, there is a need to use appropriate instruments for the measurement of textural characteristics based on food bolus properties. For instance, the viscosity of a liquid can be measured with a viscometer; the texture of a solid or gel-type food can be analyzed using a texture analyzer; the viscoelasticity of a fluid can be observed by employing a rheometer.

The properties of a food product can be measured using a dynamic rheometer. Despite the availability of precision measuring instruments for the determination of the flow behaviors of liquid food products, the development of fluid matrices with stable viscosity characteristics that last for days or weeks is still necessary to address the practical considerations for elderly care, such that long-term care workers can direct their attention to other aspects of care work.

To be able to provide proper fluid matrices with retained quality which last for days or even weeks to individuals with dysphagia, thermal processing should be imposed for sanitation considerations. Basically, food thickeners manifest as the substances capable of increasing the viscosity of fluid matrices by associating with water molecules [5,7] to form hydrogels. Pre-gelatinized tapioca starch (TS) would be a seemingly overwhelming thickener in the case of fresh preparation. However, some other thickeners, such as hydroxypropyl distarch phosphate (HDP, approved by the European Union and listed as E-1442), a modified resistant prepared from tapioca starch, or xanthan gum (XG), would also be even more preferable in terms of resistance to thermally-induced consistency alterations [11,12,13,14]. Moreover, both HDP and XG could possibly be better associated with fluid bases other than plain water due to their cross-linkages and contained ionic affinity natures [11,14], especially for those fluid bases with low pH and/or relatively high ion contents [15,16]. To date, investigations into the aforementioned flow consistency correlations amongst thickener, thermal processing level, and fluid base variations are lacking.

Therefore, the present study aimed to investigate the effects of thermal processing at temperatures of 80 and 121 °C, representing pasteurization and pressured processes, respectively, on the the flow characteristics, physical and chemical properties, and textural stability of fluid bases (distilled water—DW, sport drink—SD, and orange juice—OJ) separately thickened with TS, HDP (E-1442), and XG to provide a reference for the development of easy-to-swallow food products of stable quality.

## 2. Materials and Methods

**Figure 1:**
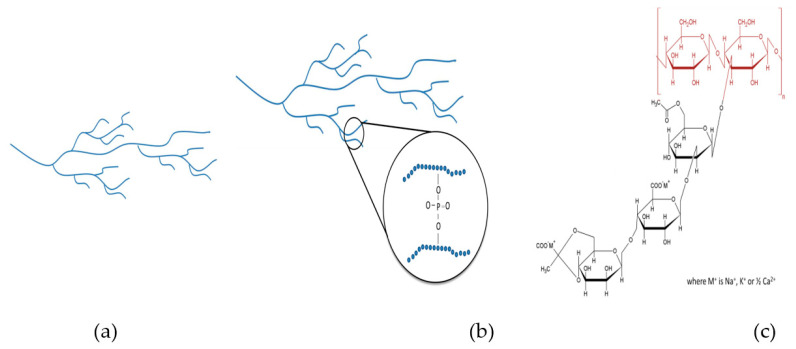
Schematic structures of (**a**) TS, (**b**) HDP/E-1442, and chemical structure of (**c**) XG. TS: tapioca starch; HDP: hydroxypropyl distarch phosphate; XG: xanthan gum.

The commercial thickeners TS, HDP, and XG used in this study were kindly donated by a local food additive agency (Kufex Corporation, Chiayi, Taiwan). Both starches (TS and HDP) originated from Thailand and have undergone a pre-gelatinization process, whereas the XG originated from France. TS is documented to possess 58 mol% unbranched amylose with an average degree of polymerization by weight of 6680 [17]. HDP is a thickener approved for use in the European Union (listed as E1442) with hydroxypropyl groups less than 7.00 g/100 g [14,18]. XG is a long-chain polysaccharide (circa 2000 kDa) with d-glucose, d-mannose, and d-glucuronic acid as building blocks (back bone) in a molecular ratio of 3:3:2 with a high number of trisaccharide side chains associated with cations of sodium, potassium, and calcium [19,20]. Figure 1 indicates the approximate structure attributes of TS, HDP, and XG. The employed fluid bases were DW, SD, and OJ. The SD (electrolyte concentration in meq/L: Na^+^ = 18.0, K^+^ = 2.4, Ca^2+^ = 1.6, Mg^2+^ = 0.8, Cl^−^ = 14.0, and PO_4_^3−^ = 0.1; calories = 27.2 per 100 g; sugar = 6.7 g per 100 g) and OJ (calories = 37.1 per 100 g; sugar = 7.9 g per 100 g; pH = 3.67) were from well-known beverage manufacturers. Initially, we identified the functional properties (solubility index—SI, swelling power—SP, water holding capacity—WHC, and oil binding capacity—OBC) of the commercial thickeners. Each thickener was incorporated into the fluid bases and formulated into a nectar-like consistency (300 ± 20 mPa.s) for the evaluation of flow and rheological behaviors. The stabilities affected by different processes (80 °C, 3 min; 80 °C, 6 min; 121 °C, 15 min) and the storage conditions were also investigated by comparing the measured apparent viscosity and textural properties before and after a 28 day storage at 4 °C.

### 2.1. Functional Properties of Thickeners

#### 2.1.1. Solubility Index and Swelling Power

The measurement of SI and SP was performed according to a method previously proposed, with modifications in sample weights and temperatures of the water bath [21,22]. Briefly, a 0.1 g (W_0_) sample was weighed into a centrifuge tube and mixed with 10 mL of DW, followed by a 30 min rotary water bath at 60, 70, 80, and 90 °C; after cooling the homogenized sample to room temperature (23 ± 2 °C), the supernatant resulting from a 20 min centrifugation at 2000× *g* was dried in an oven at 105 °C until reaching constant weight (W_sup_) within 24 h, while the sediment was weighed (W_sed_) after centrifugation without undergoing further oven drying. The SI and SP were calculated as follows:

SI (%) = (W_sup_/W_0_) × 100%; SP (g/g) = W_sed_/[W_0_ × (100% − SI)].

#### 2.1.2. Water Holding Capacity (WHC)

The WHC was measured according to a previously proposed method [22,23], with a sample weight of 0.1 g (W_0_). A total of 10 mL of DW was employed as the absorbing substrate. The sample–substrate mixture was vortexed for 1 min and remained still at 4 or 25 °C for 1 h, after which it was centrifuged at 2000× *g* for 20 min. After discarding the supernatant portion of the centrifuged mixture, the leftover aquatic counterpart was weighed as W_WH_ to determine the WHC using the following calculation:

WHC (mL/g) = (W_WH_ − W_0_)/W_0_

#### 2.1.3. Oil Binding Capacity (OBC)

The OBC was measured according to the previously proposed method [22,23], with a sample weight of 0.1 g (W_0_). The absorbing substrate employed was 10 mL of olive oil instead of DW. The sample–substrate mixture was vortexed for 1 min and remained still at 4 or 25 °C for 1 h, after which it was centrifuged at 2000× *g* for 20 min. After discarding the supernatant portion of the centrifuged mixture, the leftover oily counterpart was weighed as W_OB_, and the OHC was calculated using the following equation:

OBC (mL/g) = (W_OB_ − W_0_)/W_0_.

### 2.2. Evaluating Flow Properties of Thickened Fluid Matrices with Different Continuous Phases

#### 2.2.1. Sample Preparation for the Measurement of Apparent Viscosity

For each thickened fluid matrix (TFM), we employed the designated weight of a particular thickener (TS, HDP, or XG) as the dispersed phase and mixed with the targeted continuous phase of DW, SD, or OJ for 30 min using an electrical magnetic mixer. Thereafter, the TFM remained still at room temperature overnight to allow the TFM to become dissolved. The apparent viscosity of the TFM for each sample was measured using a viscometer (DV2 T-RV, Brookfield Asset Management Inc., Toronto, Canada) with a RV-2 spindle 60 s after reaching a shear rate of 50 s^−1^, according to the method described previously [22]. Then, the systematic apparent viscosity data as a function of the thickener weight with 1% for TS and HDP or 0.1% for XG were established.

#### 2.2.2. Flow Properties Evaluation of Thickened Fluid Matrices

Fluid apparent viscosities within a 300 ± 20 mPa.s range were prepared as test samples, followed by the shear stress vs. shear rate evaluation using a viscometer (DV2 T-RV, Brookfield Asset Management Inc., Toronto, ON, Canada) with the same spindle (RV-2) at rotating speeds of 1, 2, 5, 10, 25, 50, and 100 rpm. The resulting shear stress/shear rate curves were fitted into Herschel–Bulkley, power law, and Casson models to evaluate the corresponding flow properties according to the description of a previous study [24,25]:

Herschel–Bulkley model: σ = σ_0_ + Kγ^n^; (1)

Power law model: σ=Kγ^n^;

Casson model: σ^0.5^ = (σ_0_)^0.5^ + K_c_ γ^0.5^.

where σ: shear stress (Pa); σ_0_: yield stress (Pa); γ: shear rate (s^−1^); K: consistency index (Pa·s^n^); K_c_: Casson model parameter (Pa·s^n^); n: flow behavior index.

### 2.3. Rheological Behavior of Thermally Processed Thickened Fluid Matrices

Dynamic rheological characteristics were measured according to a previous study [26]. The experimental dynamic rheometer (AR2000ex, TA Instruments, New Castle, DE, USA) with a stainless plate 60 mm in diameter (cone angle: 1°59′20″, truncation: 53 μm) was employed to measure the strain sweep and determine the linear viscoelastic region; therefore, a strain value was chosen for time, temperature, and frequency sweeps to evaluate other rheological behaviors. The obtained storage modulus (G′) and loss modulus (G″) can also be converted into complex modulus (G*) and loss tangent (*tanδ*):

(2) $$G^{*}=\sqrt{{\left(G^{\prime }\right)}^{2}+{\left(G^{\prime \prime }\right)}^{2}};\;tan\delta =\frac{G^{\prime \prime }}{G^{\prime }}.$$

The time and temperature sweeps were performed for the heating progression from 25 to 80 °C followed by either a 3 or 6 min holding time at 80 °C, and subsequent cooling progression from 80 to 25 °C. For each heating–holding–cooling cycle, 1.9 mL of the sample was positioned at the center of the Peltier plate; a thin layer of mineral oil was applied around the sample fluid matrix, which was covered by a solvent trap to avoid water molecule evaporation. The heating and cooling rates were constrained at a 5 and −5 °C/min interval, respectively, according to a previous study [26]. After being cooled to 25 °C, the sample matrices remained still for 10 min to allow stabilization, and in sequence were subjected to frequency sweep ranging from 0.1 to 100 Hz, according to [26].

### 2.4. Flow and Textural Quality Maintenance of the Thickened Fluid Matrices

The apparent viscosity and related textural properties before and after a 28 day storage were investigated. The measurement of apparent viscosity was described in Section 2.2.1. The related textural properties were determined according to a previous method [22]. After transferring 40 mL of a given thickened fluid sample into a 50 mm diameter container, a back-extrusion disc (A/BE45, 45 mm diameter) was attached to a texture analyzer (TA-XT 2i, Stable Micro Systems, Surrey, England) equipped with a 10 kgf load cell. For evaluating the textural properties of experimental fluids, each sample was centrally positioned in the container. The disc was dipped into the sample until reaching 40% of strain, and then returned to its initial position. All progression speeds including pre-test, test, and post-test were set at 0.5 mm/s, with the trigger force of 5 gf, and the consistency and viscosity indices were obtained.

### 2.5. Statistical Analysis

The regression equations resulting from flow properties and rheological behavior were obtained using SigmaPlot^®^ version 10.0. Each experimental data set is reported as the mean value with a standard deviation based on triplicates. Independent sample *t*-test and Duncan’s multiple range test based on analysis of variance (ANOVA) were utilized to justify the significance of differences among the mean values with a 95% confidence interval using SPSS (Statistical Package for the Social Science) version 19.0.

## 3. Results and Discussion

### 3.1. Functional Properties of Thickeners

**Figure 2:**
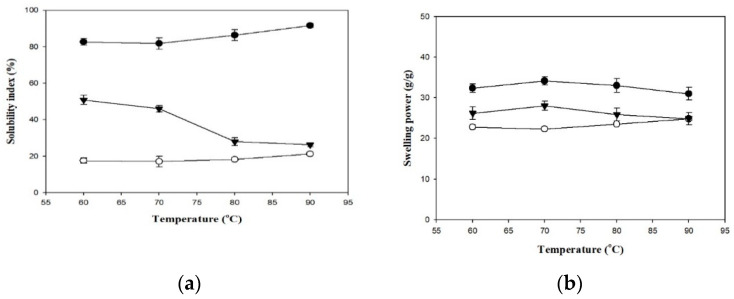
The solubility index (**a**) and swelling power (**b**) of formula thickeners at different temperatures. Tapioca starch (●); hydroxypropyl distarch phosphate (○); xanthan gum (▼). Error bars represent standard deviations. The uppercase letters are significantly different (*p* < 0.05) at the same temperature. The lowercase letters are significantly different as a function of temperature for the same thickener (*p* < 0.05).

Figure 2 presents the SIs and SPs of the employed thickeners. Generally, the SI of a hydrocolloid substance reflects the degree of dissolution of the dissolved matter in water during the swelling process. When the thickener granules are heated, water enters the network matrices and causes the particles to absorb water and swell. Differences in heating temperature also cause differences in SI. At all heating temperatures, SI was higher in the TS group (82.67–91.57%) and lower in the HDP group (17.56–21.26%), and the differences between the two groups were statistically significant (*p* < 0.05). The SI of the TS and HDP groups increased with an increase in temperature. In addition, the SI values of the TS and HDP groups were significantly higher at 90 °C compared to those at lower processing temperatures (*p* < 0.05), with the SI values being 91.57% and 21.26%, respectively.

The SP reflects the degree of swelling in the granules of the hydrocolloid substance and is a key factor that affects food quality [27]. The SP was higher in the TS group (31.01–34.14 g/g) and lower in the HDP group (22.34–24.82 g/g) at all processing temperatures, and the difference between these groups was statistically significant at 60, 70 and 80 °C (*p* < 0.05) but insignificant at 90 °C. Within each group, SP also differed with processing temperatures. SP values of the HDP group at 80 and 90 °C (23.52 g/g and 24.82 g/g, respectively) were significantly lower (*p* < 0.05) than the SP values at lower processing temperatures. In the TS group, SP initially increased and then decreased. The highest SP value was 34.14 g/g at 80 °C, but different processing temperatures did not result in significant differences in SP.

As indicated in a previous study [28], the structures of starch granules are mainly composed of radial channels formed by semi-crystalline and amorphous material. Thermal processing destroys these structures, thereby inhibiting starch swelling and delaying gelatinization. Therefore, thermal processing of starches causes a decrease in SP and a corresponding increase in SI. Previous studies have shown that the amylose and amylopectin contents of starches affect SI and SP, respectively [29]. In addition, cross-linking reinforces the bonds in starch granules, and an increase in cross-link density causes a decrease in SI and SP [30,31]. Another study indicated that the SI and SP of cross-linked oat starch decreased as the degree of cross-linking increased [32]. HDP (E-1442) is classified as modified cross-linked starch; the particular HDP used in the present study is of the tapioca origins. Its degree of cross-linkage gives a much more compact structure to constrain SP as compared to its corresponding native starch, i.e., the TS used in our formula [30]. Other reports in such light have also been well-documented towards modifications of waxy rice and waxy maize starch [33,34,35].

**Figure 3:**
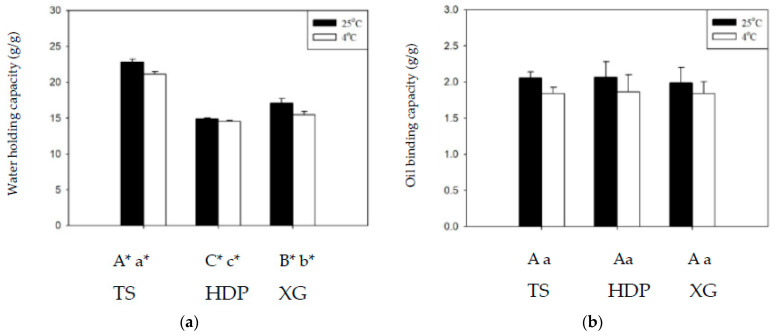
The water holding capacity (**a**) and oil binding capacity (**b**) at 4 and 25 °C for the thickeners. TS: tapioca starch; HDP: hydroxypropyl distarch phosphate; XG: xanthan gum. Error bars represent standard deviations. The uppercase letters are significantly different (*p* < 0.05) at 4 °C. The lowercase letters are significantly different (*p* < 0.05) at 25 °C. * is significant difference (*p* < 0.05) between 4 and 25 °C.

Figure 3 shows the WHC and OBC of the employed thickeners at 4 and 25 °C. Across all groups, WHC was higher at 25 °C than at 4 °C, with significant statistical differences (*p* < 0.05), whereas differences in OBC at different temperatures were not statistically significant. At 25 °C and 4 °C, the TS group exhibited the highest WHC values of 22.81 g/g and 21.12 g/g, whereas the lowest values of 14.88 g/g and 14.57 g/g, respectively, were obtained for the HDP group, with statistically significant (*p* < 0.05) differences between these groups. With regard to OBC, the HDP group had the highest values of 2.07 g/g and 1.86 g/g, and the TS group had the lowest values of 2.06 g/g and 1.84 g/g, at 25 °C and 4 °C, respectively, with statistically insignificant differences between these groups. Cross-linking modifications of starches enable the reduction in retrogradation at low temperatures, but also result in a decrease in clarity and WHC [36]. Proteins in starches affect OBC as the hydrophobic amino side chains of proteins can form bonds with lipids [27,28,29,30].

Previous research had mainly adopted SI, SP, WHC, and OBC as the functional characteristics of starches or starch-hydrocolloid gel mixtures, with few studies performing measurements on pure hydrocolloid gels. In the present study, we compared the differences between XG and the other two types of starches. The SI of the XG group decreased with an increase in temperature, with the highest value of 50.85% achieved at 60 °C. At 60, 70, and 80 °C, differences in SI between the XG and HDP groups were statistically significant (*p* < 0.05); at 90 °C, the difference in SI between the XG and TS groups was statistically significant (*p* < 0.05) but the difference with the HDP group was statistically insignificant. The SP of the XG group exhibited an initial increase and subsequent decrease as the temperature increased, reaching the highest value of 21.99 g/g at 80 °C. At 60, 70, and 80 °C, differences in SP were statistically significant between the XG and TS groups (*p* < 0.05) and statistically insignificant between the XG and HDP groups; at 90 °C, differences in SP among the three groups were not statistically significant. The WHC values of the XG group at 25 and 4 °C were significantly different from those of the TS and HDP groups (*p* < 0.05), but differences in OBC at both temperatures were not significant. In general, the values of the functional characteristics (SI, SP, WHC, and OBC) of XG were between those of TS and HDP. Our present study also indicates that XG can be considered as a refined candidate to be employed as long as the aforementioned physicochemical properties, except OBC, require minor adjustment to meet certain specifications that are not able to be achieved using HDP or TS alone.

### 3.2. Weight-Dependent Apparent Viscosity with Respect to Different Thickeners

**Figure 4:**
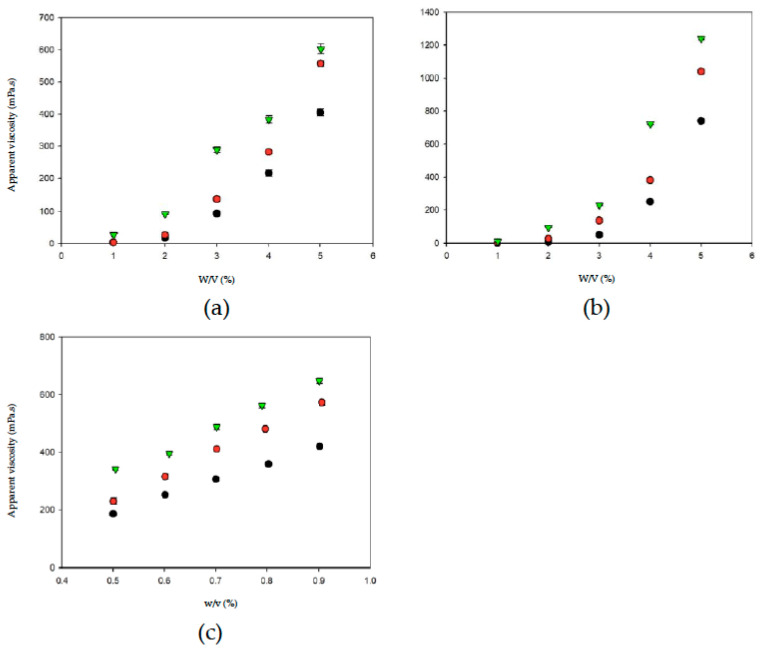
The weight-dependent (*w*) apparent viscosity with respect to thickeners incorporated into aquatic bases (*w/v*%). Water (●); sport drink (●); orange juice (▼). Error bars represent standard deviations. Thickeners: (**a**) tapioca starch; (**b**) hydroxypropyl distarch phosphate; (**c**) xanthan gum.

Thickened fluid matrices can assist in the safe consumption of liquids by dysphagia patients. Apparent viscosity may be used as a criterion for classifying and determining the stability of thickened liquids [37]. Figure 4 shows the relationships between thickener weight and apparent viscosity in different aquatic continuous phases/bases. With the same thickener weight, OJ exhibited the highest degree of thickening, followed by the SD and DW. When the thickener weight increased, the apparent viscosities of the thickened aquatic continuous phases/bases showed different tendencies of increase. The change in apparent viscosity followed a power tendency in continuous bases thickened with TS or HDP and a linear tendency in continuous bases thickened with XG, as shown in Table 1. In general, with the same thickener weight, the degree of thickening was higher in continuous bases with XG followed by HDP and TS; with the same type of thickener, the degree of thickening was higher in OJ followed by SD and DW. The results described above indicate that the apparent viscosity of food products increases with an increase in the amount of thickener used, which is consistent with the results of previous studies that investigated the viscosity stability of TS and the viscosities of aqueous XG solutions at different concentrations [38,39].

**Figure 5:**
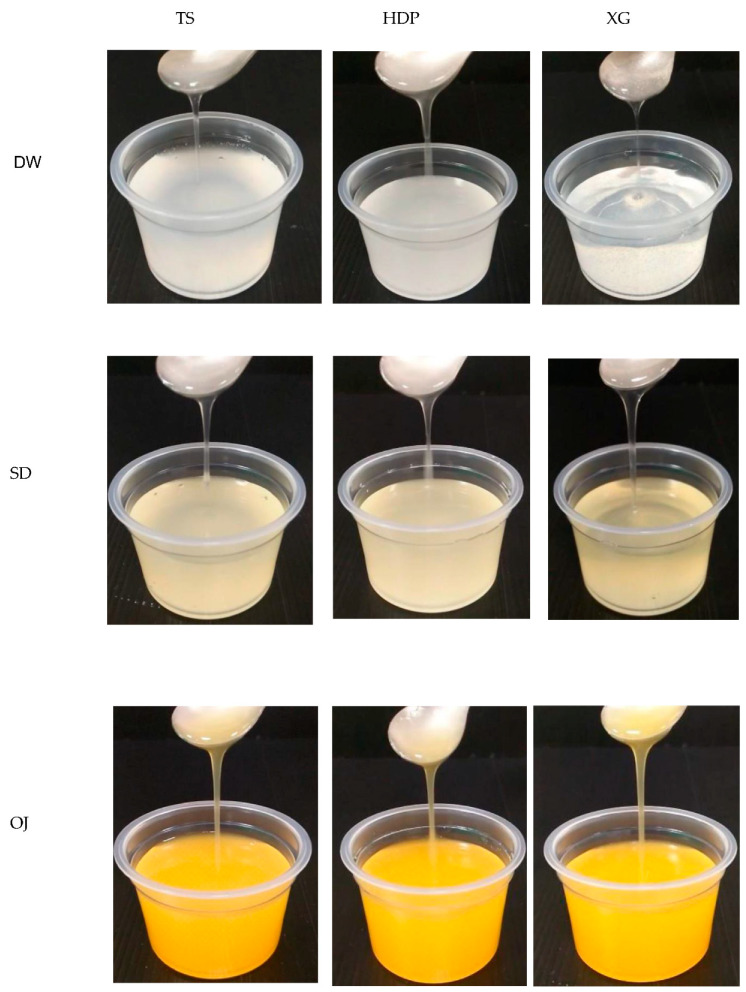
Photos of nectar-like fluids formulated by commercial thickeners associated with different aquatic bases as continuous phases.

Table 2 and Figure 5 show the formulas and appearance traits of the prepared thickened continuous bases, respectively. We have previously reported that 10.40 g of germ rice flour and a proximal amount of XG per 100 mL of distilled water achieves a nectar-like consistency [22]. The present results indicate that the TS sample starch used is in a more condensed form (3.374–4.505 g) in terms of capability to associate water molecules for consistency promotion. The results of a survey on the use of thickened liquids in long-term care facilities showed that, among the residents receiving thickened liquids, approximately 60% received nectar-thick liquids, 33% received honey-thick liquids, and 6% received pudding-thick liquids [40]. Therefore, the apparent viscosity of the thickened continuous bases in this study was constrained at 300 ± 20 mPa.s to achieve a nectar-like consistency for ongoing experimentations as detection limits of facilitate apparatus and feasibility of product attributes.

### 3.3. Flow Properties of Formulated Nectar-Like Fluids

Previous studies have shown that fluid characteristic parameters such as flow behavior index (n), consistency index (K), and yield index (σ_0_) are related to the swallowing process [24,41,42]. In particular, K is related to the speed of the food bolus movement, n is related to the oral sensation of the food bolus smoothness, and σ_0_ is related to the ease of swallowing of food boluses [43,44].

**Figure 6:**
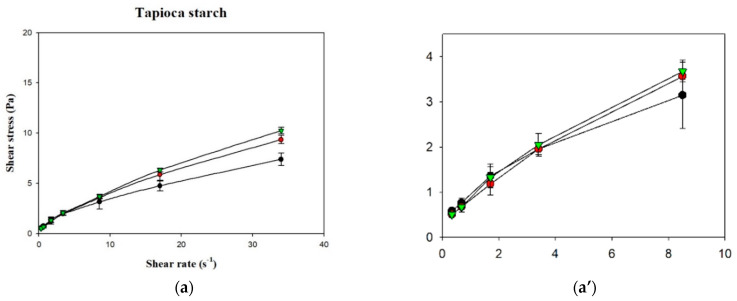

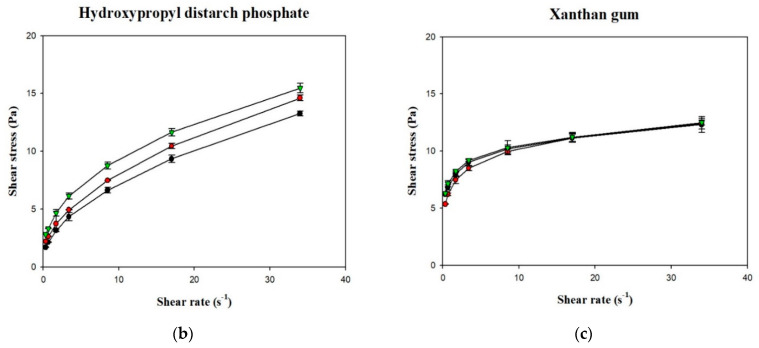
Shear rate-shear stress profile of different thickened beverages. Water (●); sport drink (●); orange juice (▼). Error bars represent standard deviations. Thickeners: (**a**) tapioca starch with a partial magnification (**a’**); (**b**) hydroxypropyl distarch phosphate; (**c**) xanthan gum.

Figure 6 shows the shear stress-shear rate curves of the various samples plotted using measurements obtained with a rotational viscometer. It can be seen that the curves for the various groups were non-linear with an intercept on the vertical axis (shear stress), which indicates that the liquids were non-Newtonian fluids with a yield stress. Using the Herschel–Bulkley model, the values of n, K, and σ_0_ were calculated, as shown in Table 3. Although R^2^ of the various groups ranged from 0.9987 to 0.9999, the groups in which XG was used as the thickener had σ_0_ values <0, which shows that the Herschel–Bulkley model is not applicable for XG-thickened continuous bases. Therefore, by adopting the method proposed by Yoon and Yoo [10], the power law was used for the calculation of n and K and the Casson model was used for the calculation of σ_0_, as shown in Table 4. It was found that the K and σ_0_ values were higher in continuous bases thickened with XG and lower in continuous bases thickened with HDP. The opposite tendency was observed with the n values, which were higher in continuous bases thickened with TS followed by continuous bases thickened with HDP and XG. With the same beverage base, the K, n, and σ_0_ values significantly differed among the various groups (*p* < 0.05). When TS was used as the thickener, the DW group had the highest K and σ_0_ values of 0.9334 Pa·s^n^ and 0.4047 Pa, respectively, and the lowest n value of 0.5835. K was significantly higher in the DW group compared to the other two groups (*p* < 0.05), and σ_0_ and n significantly differed among the three groups (*p* < 0.05). When HDP was used as the thickener, the OJ group had the highest K and σ_0_ values of 3.8295 Pa·s^n^ and 2.4338 Pa, respectively, and the lowest n value of 0.3940. The n value of the OJ group was significantly lower than that of the other two groups (*p* < 0.05), and σ_0_ and K significantly differed among the three groups (*p* < 0.05). When XG was used as the thickener, the OJ group exhibited the highest K and σ_0_ values of 7.5538 Pa·s^n^ and 6.6054 Pa, respectively, and the lowest n value of 0.1432. The SD group had a significantly lower σ_0_ value (*p* < 0.05) and a significantly higher n value compared with the other two groups, and K was significantly different among the various groups (*p* < 0.05).

Fluids with n < 1 exhibit shear-thinning behavior. In the present study, the n values of the various thickened continuous bases ranged from 0.1432 to 0.7048, indicating that thickened continuous bases prepared by thickening DW, SD, and OJ with TS, HDP and XG were non-Newtonian fluids with shear-thinning characteristics. R^2^ ranged from 0.9970 to 0.9997 for K and n values obtained using the power law and from 0.9120 to 0.9958 for σ_0_ values obtained using the Casson model, which shows that the power law and Casson models could adequately describe the fluid characteristics of the thickened continuous bases prepared in this study.

In addition, it can be observed that K was positively correlated with σ_0_ and negatively correlated with n, which is similar to the fluid characteristics of the TS matrices investigated by Chen and Ramaswamy [25]; they measured the flow curves of native tapioca starch using a different brand name of rotational viscometer (Haake Model RV20) and an MV1 spindle considered comparable to our present study. The concentration used in their study is 4% *w*/*v*, which is close to our TS-containing sample incorporated into DW (4.505 g/100 mL); however, our TS is a commercial product which had been pre-gelatinized. Therefore, their experimental setup involved the hydrothermal cooking process up to 40 min for modeling the flow curves. The models used in their study are identical to our study. As for the Herschel–Bulkley model, our TS–DW matrices show a good agreement with their time-dependent modeling results: K ranging 0.64–1.08, n ranging 0.42–0.78; however, our σ_0_ (0.2328) falls beyond their reported lower range (0.41–0.95). As aforementioned, yield index, σ_0,_ is related to the ease of swallowing of food boluses [43,44]. The commercial TS we employed would be better suited for dysphagia-friendly application. We also noted that the R^2^ values studied by Chen and Ramaswamy [25], ranging 0.95–0.99, compared to our values ranging 0.9987–0.999; nevertheless, there should not be a clue of better regression results of ours because their experimental framework regarding TS flow curve determination facilitated a more stretched shear rate range up to 200 s^−1^.

These results are also in agreement with the results of a study on the fluid characteristics of thickened infant formula prepared with commercial thickeners [10]. Yoon and Yoo [10] reported the rheological behavior of thickened infant formula prepared with XG for dysphasia infants; their formulated matrices contained ions of calcium, phosphate, magnesium, iron, zinc, copper, and iodine with an XG-based concentration of at least 1%, which is greater than our inclusive concentration (0.467–0.694%). We noted that they have also fitted to the power law and Casson model, and the fitness index of R^2^ was reported to be over 0.95. The flow behavior index modeled by power law of the 1% combined thickener-base containing XG, guar gum, and dextrin (ratio not revealed) gives a considerable low value of 0.24, which is comparable to our XG-containing fluid matrices regardless of the use of DW, SD, or OJ bases with values ranging 0.14–0.18, an indication of better smoothness of mouthfeel.

### 3.4. Rheological Behaviors of Formulated Nectar-Like Fluids

**Figure 7:**
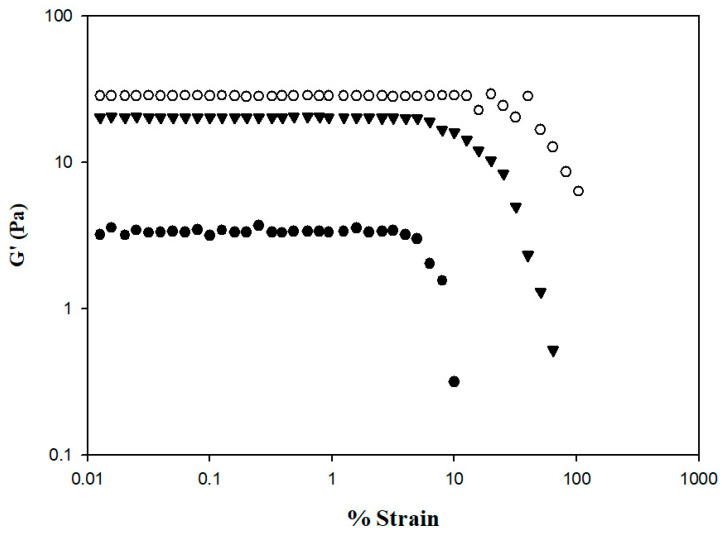
Storage modulus % strain profile of thickened SD by incorporating different commercial thickeners. Tapioca starch (●); hydroxypropyl distarch phosphate (○); xanthan gum (▼).

The rheological behaviors of the samples were analyzed using a dynamic rheometer, and the analysis was carefully performed within the linear viscoelastic range to ensure that sample structures were not destroyed by the applied stress or strain. Figure 7 shows the changes in storage modulus (G′) from 0.01 to 100% strain. At the same % strain value, HDP-thickened SD exhibited the highest G′ value, followed by the XG- and TS-thickened SD. The G′ of the TS-thickened liquid exhibited a sharp decrease starting from a % strain value of approximately 4.99; a similar tendency was observed in the XG-thickened liquid starting from a % strain value of approximately 6.32. The G′ of the HDP-thickened liquid started to fluctuate at a % strain value of approximately 15.85 and in sequence decreased, indicating that the linear viscoelastic limit had been reached. Therefore, the subsequent temperature, time, and frequency sweeps for viscoelasticity measurements of samples were performed at 0.1% strain. Bi et al. have reported a strain sweep case of 0.25% *w*/*v* XG (G810381, USP grade) that allows us to make it possible as good reference [45]; the linear viscoelastic range is between % strain of 0.1 up to certain values between 1–10 with G′ falling between 10–100. The linear viscoelastic range of our XG–DS matrices is comparable to this previous study. We would like to remark that the rheological behavior of XG can be varied from one commercial source to another, even though their molecular weight is stated somewhat between 800–1000k Dalton (E-415); it is also necessary to report that in the case of Bi et al., deionized water was used, whereas we used distilled water instead. Therefore, as discussed, we would consider and be confident that the ongoing rheological behaviors we conducted should be considerably reliable.

#### 3.4.1. Behaviors Affected by Thermal and Possible Mechanical Processes

**Figure 8:**
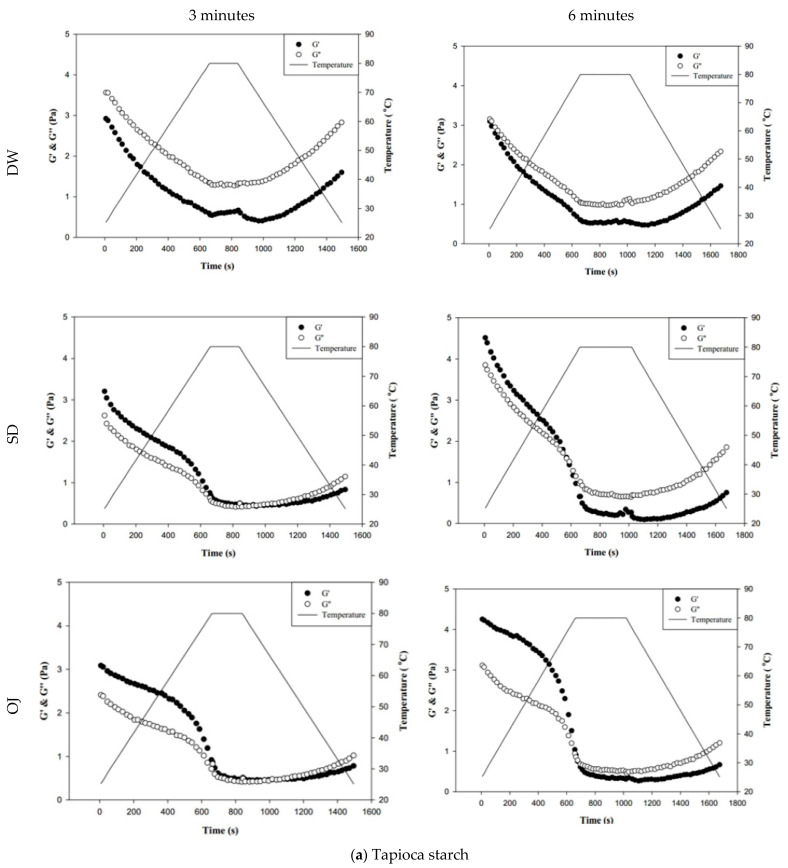

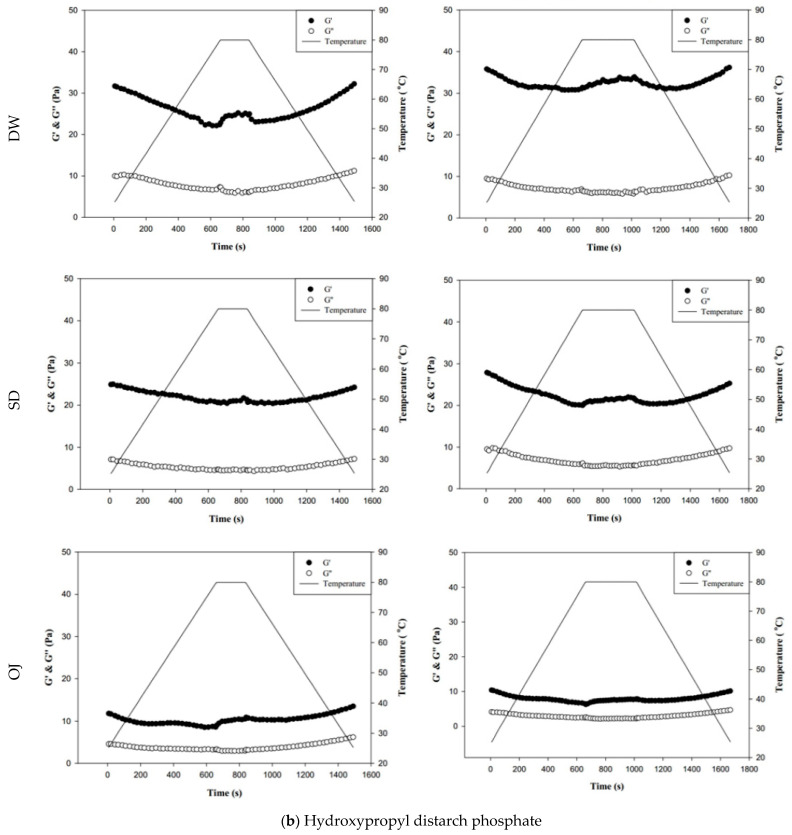

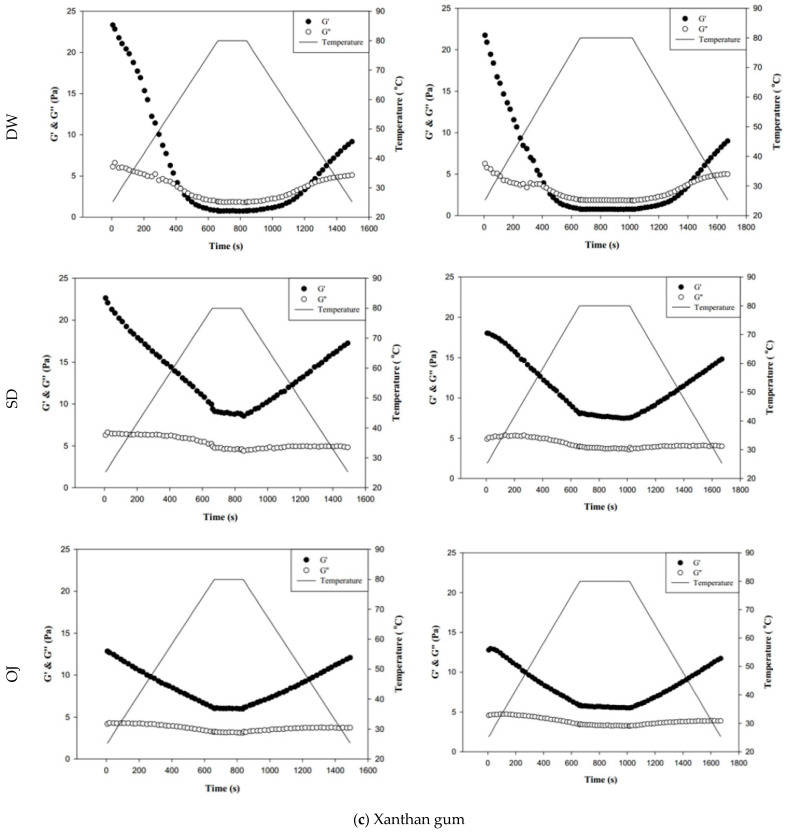
Storage modulus (G′) and loss modulus (G″) of tapioca starch (**a**), hydroxypropyl distarch phosphate (**b**), and xanthan gum (**c**) with different aquatic contiouous bases and holding times. DW: distilled water; SD: sport drink; OJ: orange juice; 3m: 3 minutes; 6m: 6 minutes. (●): G′; (o): G″; (—): temperature. (▼), and (▼) are signifcantly different at 5% and 1% levels, respectively, between G_initial_ and G_end_ using *t*-test. For tapioca starch, G′ with▼: DW-3m; G″ with ▼: SD-6m, and OJ-6m; G′ with ▼: SD-3m, SD-6m, and OJ-6m; G″ with▼: DW-6m, SD-3m, and OJ-3m. For, Hydroxypropyl distarch phosphate. G′ with▼: SD-6m. For xanthan gum. G″ with ▼: SD-6m; G′ with ▼: DW-3m, and DW-6m.

Figure 8 shows the changes in fluid properties of the various thickened continuous bases during thermal processing; the significances between the corresponding G′_initial_ and G′_end_ values, and the G″_initial_ and G″_end_ values of the thickened fluid matrices are indicated by the colored triangles in Figure 8. Figure 8-a indicates the changes in fluid properties of TS-thickened continuous bases during thermal processing. When DW was used as the fluid base, the G′_end_ and G″_end_ values of the TS-thickened liquid were lower than the G′_initial_ and G″_initial_ values, which indicates that the thermal processing decreased the elastic and viscous characteristics of the thickened liquid. The G′_end_ values of both the 3 min and 6 min processing groups were significantly lower than the corresponding G′_initial_ values (*p* < 0.05), but there were no significant differences in G″_end_ and G″_initial_. In addition, G″ was always higher than G′ during thermal processing, which indicates viscous characteristics during processing. When SD was used as the fluid base, the G′_end_ and G″_end_ values of the TS-thickened liquid were significantly lower than the G′_initial_ and G″_initial_ values. The G′ values of both the 3 min and 6 min processing groups were higher than the corresponding G″ values at the initial stage, indicating elastic characteristics. During the late stage of processing, the G″ values were higher than the G′ values, which indicates viscous characteristics. Therefore, it can be deduced that phase transition occurred during thermal processing, as shown in Figure 8. When OJ was used as the fluid base, the G′_end_ and G″_end_ values of the TS-thickened liquid were significantly lower than the G′_initial_ and G″_initial_ values (*p* < 0.05). The fluid properties of the thickened OJ showed a tendency similar to that of thickened SD, with the G′ values of both the 3 min and 6 min processing groups higher than the corresponding G″ values at the initial stage and the G″ values higher than the G′ values after the phase transition from elastic to viscous characteristics.

Figure 8b indicates the changes in fluid properties of HDP-thickened continuous bases during thermal processing. The initial and final values of G′ and G″ of the HDP-thickened continuous bases did not differ significantly; the same was observed in the 3 min and 6 min processing groups. During thermal processing, the G′ values of both the 3 min and 6 min processing groups were higher than the corresponding G″ values, indicating that the fluids did not undergo phase transition and displayed elastic characteristics. Figure 8c shows the changes in fluid properties of XG-thickened continuous bases during thermal processing. When DW was used as the fluid base, the G′_end_ values of both the 3 min processing and 6 min processing groups were significantly lower than the corresponding G′_initial_ values (*p* < 0.05), whereas G″_end_ and G″_initial_ did not differ significantly. Two phase transitions occurred in both the 3 min and 6 min processing groups during thermal processing. In the 3 min processing group, the first phase transition occurred when the temperature was increased to approximately 60 °C. Thereafter, G′ gradually decreased and became lower than G″, and the fluid exhibited a transition from elastic to viscous characteristics. The second phase transition occurred when the temperature was decreased to approximately 50 °C. G′ showed a gradual increase and became higher than G″, and the fluid exhibited a transition from viscous to elastic characteristics. Similar tendencies were observed in the 6 min processing group, with the first and second phase transitions occurring when the temperature was increased to 60 °C and decreased to approximately 47 °C, respectively. When SD was used as the fluid base, the G′_end_ and G″_end_ values of the 3 min processing group were significantly lower than the G′_initial_ and G″_initial_ values (*p* < 0.05), respectively, whereas the differences in values of the 6 min processing group were not significant. The fluids of both thermal processing groups did not undergo phase transition and had higher G′ values than G″ values, indicating elastic characteristics during thermal processing. When OJ was used as the fluid base, the G′_end_ and G″_end_ values of both the 3 min and 6 min processing groups were lower than the corresponding G′_initial_ and G″_initial_ values, but the differences were not statistically significant. The fluids of both thermal processing groups did not undergo phase transition and had higher G′ values than G″ values, indicating elastic characteristics during thermal processing.

Jyothi, Moorthy and Rajasekharan have reported pasting behaviors of cassava (tapioca) starch in native form and cross-linked form, which resembles HDP of tapioca origin used in our experiments, using a rapid visco-analyzer [30]. They prepared 10% *w*/*v* starchy slurry by heating from 50 to 95 °C with a 12 °C/min temperature increasing rate at 160 rpm rotor speed followed by a holding time of 2 min at 95 °C. The cooling stage is the reversed analogue (−12 °C/min). Their reported RVA (rapid visco analyzer) viscosity patterns of TS–DW and HDP–DW also reveal the observable “dips” during the holding period. The cross-linked tapioca starch (analogue to our HDP) could result in an almost 4-fold increase of the measured viscosity value as compared to the native starch. These observations make our rheological measurements somewhat sustained in a solid base.

**Figure 9:**
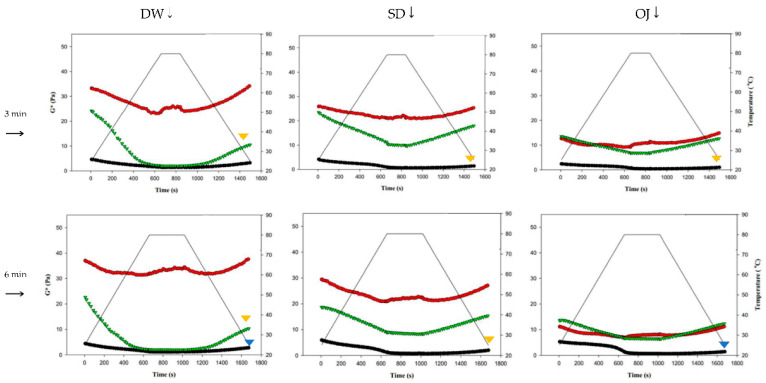
Complex moduli (G*) of different combinations of holding times, commercial thickeners, and aquatic continuous phases. DW: distilled water; SD: sport drink; OJ: orange juice; TS: tapioca starch; HDP: hydroxypropyl distarch phosphate; XG: xanthan gum. (▼) and (▼) are signifcantly different at 5% and 1% levels, respectively, between G_initial_ and G_end_ using *t*-test._._ (●) = TS; (●) = HDP; (●) = XG.

Figure 9 shows the changes in complex modulus (G*) during thermal processing in groups with different combinations of fluid base, thickener, and holding time. The colored triangles indicate significances between G* at the start (G*_initial_) and end points (G*_end_). When TS was used as the thickener, G*_end_ was significantly lower than G*_initial_ in almost all of the 3 min and 6 min processing groups (*p* < 0.05), with highly significant differences in the sugar-containing acidic beverages (SD and OJ) (*p* < 0.01). When HDP was used as the thickener, there were no significant differences between G*_initial_ and G*_end_ in all groups; when XG was used as the thickener, the G*_end_ values of the 3 min and 6 min processing groups were significantly lower than the corresponding G*_initial_ values (*p* < 0.05) for thickened DW, but did not differ significantly for the thickened SD and OJ samples. Therefore, when HDP was used as the thickener, product quality could be maintained after thermal processing as the fluid properties of the thickened liquid were not altered by heat. With TS used as the thickener, the fluid properties of the thickened liquid were altered by thermal processing. In addition, it was also found that the effects of thermal processing on product quality were more pronounced.

The viscoelasticity of starches is determined by factors such as starch granules’ soluble matter content, morphology, size, degree of swelling, and complexes formed with other components (e.g., lipid–amylose) [46]. Gels formed from amylose tend to be elastic, whereas gels formed from amylopectin tend to be viscous. Both amylose and amylopectin swell and decompose upon heating, which causes a reduction in viscoelasticity [47]. As starch molecules swell, melting of the crystalline regions disrupts the gel structure, thereby causing the softening or breakage of starch granules. With the loss of interactions among granules, the network structure is destroyed, resulting in a decrease in G′ [47,48,49]. Chemical modifications can lead to considerable changes in viscoelasticity; studies have shown that acetylated, hydroxypropylated and cross-linked starches have significantly higher G′ and G″ values than natural starches [50,51].

#### 3.4.2. Viscoelastic Characteristics

Table 5 shows the frequency sweep results described using the equation G′ = kw^n^. As the 3 min and 6 min processing groups for TS- and XG-thickened DW and the 6 min processing group of TS-thickened SD exhibited unstable G′ values when sweeping was performed at frequencies >1 Hz, results within the frequency range of 0.1–1 Hz were displayed for these groups. The R^2^ of G′ and G″ ranged from 0.9009 to 0.9984 and 0.9417 to 0.9995, respectively, indicating that the viscoelasticity of the thickened continuous bases obtained by the frequency sweep could be adequately described by G′ = kw^n^.

**Figure 10:**
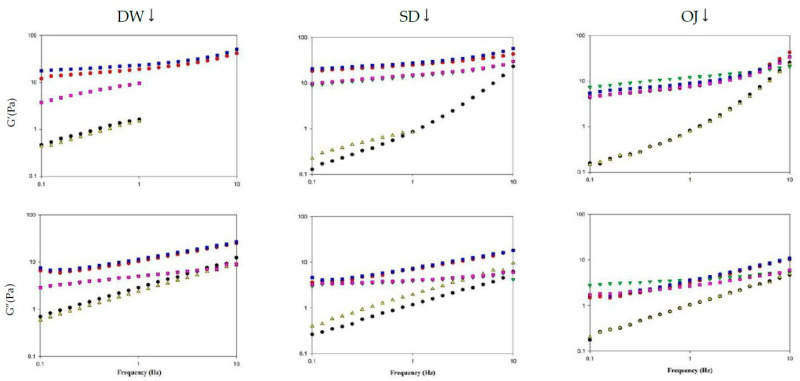
Frequency sweep of different thickened fluid matrices. TS: tapioca starch; HDP: hydroxypropyl distarch phosphate; XG: xanthan gum; 3m: 3 min; 6m: 6 min. (●) = TS-3m; (●) = HDP-3m; (▼) = XG-3m; (▲) = TS-6m; (■) = HDP-6m; (■) = XG-6m.

The viscoelasticity of thickened continuous bases can be determined by the frequency sweep test, and the fluids can be classified into dilute and concentrated solutions and gels based on the resultant spectra [52]. Figure 10 shows the spectra obtained from frequency sweeps of the various thickened continuous bases. When DW was used as the fluid base, the G′ and G″ values of the various processing groups increased with frequency and were higher in the HDP-thickened liquids followed by the XG-thickened liquids. TS-thickened DW had lower G′ values than G″ values and exhibited viscous characteristics. In contrast, HDP- or XG-thickened DW had higher G′ values than G″ values and exhibited elastic characteristics. As the tendencies of G′ and G″ indicated the presence of a weak gel network structure, the fluids were classified as weak gels.

When SD was used as the fluid base, the G′ and G″ values of the various processing groups increased with frequency and were higher in the HDP-thickened liquids followed by the XG- and TS-thickened liquids. HDP- and XG-thickened SD had higher G′ values than G″ values and exhibited elastic characteristics; the tendencies of G′ and G″ also indicated the presence of a weak gel network structure. When TS was used as the thickener, the 3 min processing group had lower G′ values than G″ values and exhibited viscous characteristics at low frequencies. At a frequency of 1.9950 Hz, the G′ and G″ values were 1.8677 Pa and 1.8607 Pa, respectively, and G′ started to exceed G″. Elastic characteristics were observed at high frequencies and the fluids were classified as concentrated solutions based on the G′ and G″ tendencies.

When OJ was used as the fluid base, the G′ and G″ values of the various groups increased with an increase in frequency. At low frequencies, the 3 min and 6 min processing groups for HDP-thickened OJ had lower G′ values than G″ values and exhibited viscous characteristics. When the frequency was increased to 1.9950 Hz, phase transition occurred in both groups and G′ started to exceed G″, with G′ values of 1.8173 and 1.6980 Pa for the 3 min and 6 min groups, and G″ values of 1.6060 and 1.5963 Pa for the 3 min and 6 min groups, respectively. Elastic characteristics were observed at high frequencies and the fluids were classified as concentrated solutions based on the G′ and G″ tendencies.

Table 6 shows the viscoelasticity parameters of the thickened continuous bases formulated with different thickeners and continuous bases, measured at a frequency of 1 Hz. When DW was used as the fluid base, the 3 min and 6 min processing groups of HDP-thickened DW had G′ values of 34.0733 Pa and 46.3567 Pa, respectively, and G″ values of 10.2430 Pa and 11.9710 Pa, respectively, with G′ and G″ being significantly higher compared with the other processing groups (*p* < 0.05). The 3 min and 6 min processing groups of TS-thickened DW had the highest *tanδ* values of 1.75 and 1.56, respectively, which were significantly higher compared with other groups (*p* < 0.05). When SD was used as the fluid base, the 3 min and 6 min processing groups of HDP-thickened SD had G′ values of 24.98 Pa and 27.59 Pa, respectively, and G″ values of 6.98 Pa and 7.38 Pa, respectively, with G′ and G″ significantly higher compared with the TS-thickened SD groups (*p* < 0.05). The G′ and G″ values of XG-thickened SD did not differ significantly compared with the other processing groups. The 6 min processing group of TS-thickened SD had the highest *tanδ* value of 2.40, which was significantly higher compared to that of the other processing groups (*p* < 0.05). When OJ was used as the fluid base, the 3 min and 6 min processing groups of TS-thickened OJ had G′ values of 0.82 Pa and 0.80 Pa, respectively, and G″ values of 1.03 Pa and 1.03 Pa, respectively, which were significantly lower compared with the other processing groups (*p* < 0.05). In addition, the 3 min and 6 min processing groups of TS-thickened OJ had the highest *tanδ* values of 1.26 and 1.30, respectively, which were significantly higher than those of the other processing groups (*p* < 0.05).

In general, the HDP-thickened liquids exhibited relatively high G′ and G″ values, and the TS-thickened liquids had relatively low *tanδ* values. *tanδ* can be used as the basis for determining the viscoelasticity of fluids, as *tanδ* >1 indicates G′ < G″ and *tanδ* < 1 indicates G′ > G″. When TS was used as the thickener, the *tanδ* values of the various thickened continuous bases ranged from 1.26 to 2.40, which indicated that G′ < G″; when HDP was used as the thickener, the tanδ values of the various thickened continuous bases ranged from 0.25 to 0.42, which indicated that G′ > G″; when XG was used as the thickener, the *tanδ* values of the various thickened continuous bases ranged from 0.27 to 0.52, which indicated that G′ > G″. Therefore, at a frequency of 1 Hz, TS-thickened continuous bases exhibited viscous characteristics and behaved like viscous materials, whereas HDP- and XG-thickened materials exhibited elastic characteristics and behaved like elastic materials.

### 3.5. Variations of Flow and Textural Parameters with Respect to Maintenance of Quality

The effects of different thermal processes on the apparent viscosities and textural parameters (viscosity and consistency indexes) of the various thickened continuous bases were investigated, and the changes after 28 days of storage were compared. Table 7 and Table 8 show the comparison of the parameter values on the day of processing and after 28 days of storage at 4 °C. The various processing temperatures promoted different effects on apparent viscosity, with statistically significant (*p* < 0.05) differences between the various processing and control groups. In particular, processing at 121 °C for 15 min provided the greatest effect (Table 8), which resulted in percentage changes of −28.12% to −98.14%. Apparent viscosity obtained with processing at 80 °C for 3 min was lower than that obtained with processing at 80 °C for 6 min in TS-thickened DW, HDP-thickened DW, and XG-thickened OJ, but similar results were not observed in other thickened continuous bases. This may be attributed to the lower degree of gelatinization with 3 min of processing than that with 6 min of processing in the previously mentioned groups.

The consistency index values of the various processing groups did not differ significantly from those of the control groups and exhibited a similar tendency to firmness (data not shown). This is due to the fact that firmness, which represents the maximum positive force in textural analysis, is related to the consistency index, which is the positive peak area under the force-time curve [40]. For TS-thickened DW, TS-thickened SD, HDP-thickened SD, and XG-thickened SD, the viscosity index values obtained with processing at 80 °C for 3 min and 6 min were significantly different from the values of the corresponding control groups (*p* < 0.05) and exhibited a similar tendency to cohesiveness (data not shown). This is due to the fact that cohesiveness, which represents the maximum negative force in textural analysis, is related to the viscosity index, which is the negative peak area under the force-time curve [53].

The results described above indicate that, among the various thickened continuous bases, those prepared using SD as the fluid base exhibited considerable changes in apparent viscosity and textural parameters after thermal processing. Continuous bases thickened with XG exhibited relatively stable properties after thermal processing compared with continuous bases thickened with other thickeners. Apparent viscosity showed more significant changes (*p* < 0.05) after thermal processing compared with the other parameters. Among the various processing conditions, a temperature of 121 °C and a hold time of 15 min promoted the greatest effects on the physicochemical properties and textures of thickened continuous bases. After processing at 121 °C for 15 min, the apparent viscosities of most of the thickened continuous bases were below 50 mP.s. As liquids were classified as thin liquids and unsuitable for consumption by dysphagic patients, they were excluded from the subsequent storage experiment. In addition, it can be observed that the apparent viscosities provided by the various thickeners were similar, but differences were observed in the various textural parameters. This indicates that the textural parameters could be more related to the type of thickened liquid, but such differences may also be caused by differences in the methods used for textural analysis or the speed at which the compression plate was raised or lowered.

When starch is heated in hot water, thermal energy promotes the entry of water into its amorphous regions. With sustained heating, the hydrogen bonds of the crystalline regions are ruptured, enabling the entry of water into the crystalline regions. The destruction of crystalline regions during the gelatinization process leads to a gradual loss of birefringence and the formation of loose granular structures. When the starch granules imbibe water and swell, their density decreases and granule size increases, causing an increase in internal friction and resulting in increased viscosity [41,54]. The types and contents of sugar in continuous bases also affect the viscosities and textures of the thickened continuous bases. In previous studies on wheat starch-milk-sugar systems (WMS) and corn starch-milk-sugar systems (CMS), it was reported that the highest increase in the viscosities of WMS and CMS was obtained with the addition of fructose and glucose, respectively [55,56,57]. In addition, the presence of an acidic environment leads to partial hydrolysis of molecules on the surface of starch granules and increases the hygroscopicity of starch granule surfaces, thereby increasing the viscosity. However, excessive hydrolysis can also cause starch granules to rupture, which results in a decrease in the gelation ability [58].

Our results indicated that the apparent viscosity changed with storage time. TS- or XG-thickened continuous bases exhibited gradual changes in apparent viscosity, which ranged from 81.73 mPa.s to 300.33 mPa.scP, and were classified as nectar-like liquids. In contrast, HDP-thickened continuous bases exhibited relatively unstable apparent viscosities. A comparison of the apparent viscosities on day 28 and day 0 indicated that HDP- and TS-thickened continuous bases exhibited the highest and lowest values, respectively. Among the groups subjected to processing at 80 °C for 3 min, the TS-thickened continuous bases had the highest decrease in apparent viscosity (−25.31%), whereas the HDP-thickened OJ exhibited the highest increase (+25.84%). Among the groups subjected to processing at 80 °C for 6 min, the TS-thickened continuous bases had the highest decrease in apparent viscosity (−30.99%), whereas the HDP-thickened DW exhibited the highest increase (+19.62%). In general, the various thickened continuous bases only exhibited gradual changes in consistency index over the storage period. Among the various thickened DW samples, the HDP group exhibited the highest consistency index, followed by the XG group. For the thickened SD and OJ samples, the XG group had the highest consistency index within groups of the same fluid base, followed by the HDP and TS groups. The thickened continuous bases also exhibited gradual changes in viscosity index over the storage period. Similarly, the HDP group had the highest viscosity index among the various thickened DW samples, followed by the XG group. For the thickened SD and OJ samples, the XG group had the highest consistency index within groups of the same fluid base, followed by the HDP and TS groups.

The recruited thickeners, TS, HDP, and XG, in our study could result in different related properties while being prepared as thickened fluid matrices; in this light, we provide functional property data (SI, SP, WHC, and OBC) as well as weight-dependent apparent viscosity to different thickeners. Amongst the recruited thickeners, HDP is classified as a type IV resistant starch manifested as a functional ingredient with intra-molecule cross-linked modification to reduced glycemic response compared to digestible carbohydrate, consequently having resulted in an approved European Union health claim or elsewhere in the world [59]. Our sample OJ is often referred to as orange-flavored cordial; there has already been a solid rheological report regarding a raspberry flavored cordial; however, their sample fluid matrices were prepared by manual mixing with a spatula until homogenous as a fresh-preparation scenario without further thermal processing [16]. Dynamic rheological behaviors with respect to both cases of 80 °C and holding for 3 and 6 min in the present study can be considered as the thermal pasteurization practice for packaged fluid matrices being accommodated in containers with different critical length associated with thermal penetration; furthermore, the dynamic oscillation might as well be a related pipeline conveying or pumping progression in an aseptic package system as the shear force somehow theoretically attributing viscoelastic change of the prepared fluid matrices. Finally, we would like to make a humble note that video-fluoroscopy is commonly employed to diagnose dysphagia severity and evaluate the effectiveness of texture or viscoelasticity modification with respect to confirming safe swallowing progression in different liquid consistencies [60]; therefore, such videoscope technology is to be introduced in our future study for ensuring formulas possessing dysphagia-friendly potential.

## 4. Conclusions

TS is considered to be a good thickening candidate for instant consumption with respect to solubility, WHC, and OHC. The flow behaviors of all thickened fluid matrices with nectar-like consistency adequately fit the power law and Casson models, regardless of the variety of thickeners, and these thickened fluid matrices can be categorized as non-Newtonian fluids with a yield stress that evidence shear-thinning characteristics; among the thickeners used in the present study, XG appears to provide a higher yield stress and consistency index, but a lower flow behavior index than the other starch thickeners analyzed. Thickened fluids with an OJ base required the least amount of thickeners to achieve the nectar-like consistency for dysphagia-friendly formulation. Intensive thermal process (121℃, 15 min) is unfavorable for consistency/viscosity retention characteristics. Thermal processing causes pronounced elastic characteristics when employing XG or HDP as thickeners. Fluid matrices thickened by HDP that had undergone 80 ℃ thermal processes resulted in the best stability regarding flow properties for a 28 day storage period at 4 ℃, which could potentially relieve nursing professionals in health care facilities in the preparation of liquids for individuals with dysphagia.

## Figures and Tables

**Table 1:** Correlations between apparent viscosity (y with unit of mPa.s) and combinations of thickeners and aquatic bases.

Thickener	Base	Equation Type	Regression Equation	R^2^
TS	DW	Power law	y = 2.924·x^3.0551^	0.9988
	SD	Power law	y = 3.2471·x^3.2267^	0.9986
	OJ	Power law	y = 25.477·x^1.959^	0.9846
HDP	DW	Power law	y = 1.1044·x^3.8583^	0.9996
	SD	Power law	y = 2.8921·x^3.5511^	0.9989
	OJ	Power law	y = 10.456·x^2.9814^	0.9929
XG	DW	Linear	y = 573.38·x − 97.869	0.9983
	SD	Linear	y = 848.42·x − 193.29	0.9987
	OJ	Linear	y = 800.29·x − 73.941	0.9922

DW: distilled water; SD: sport drink; OJ: orange juice; TS: tapioca starch; HDP: hydroxypropyl distarch phosphate; XG: xanthan gum.

**Table 2:** Formula of thickened nectar-like fluids *.

Food Thickener (g)	Base			Viscosity
	Distilled Water	Sport Drink	Orange Juice	
Tapioca starch	4.505	4.061	3.374	300 ± 20 mPa.s
Hydroxypropyl distarch phosphate	4.167	3.752	3.032	
Xanthan gum	0.694	0.581	0.467	

* The thickened fluids were prepared by incorporating different weight of formula thickeners per 100 milliliters of aquatic base as continuous phase.

**Table 3:** Summary of the flow properties parameter of regression output of Herschel–Bulkley model for thickened beverages made from different commercial thickeners and fluid bases *.

Base	Thickener	σ_0_ (Pa)	*K* (Pa·s^n^)	*n*	R^2^
Distilled water	TS	0.2328 ± 0.0017	0.7576 ± 0.0100	0.6356 ± 0.0005	0.9992
	HDP	0.5835 ± 0.0025	1.9413 ± 0.0086	0.5321 ± 0.0031	0.9999
	XG	−6.1593 ± 0.0703	13.5273 ± 0.0445	0.0883 ± 0.0012	0.9989
Sport drink	TS	0.0951 ± 0.0046	0.7862 ± 0.0052	0.6999 ± 0.0017	0.9998
	HDP	1.0089 ± 0.0875	2.0507 ± 0.0610	0.5373 ± 0.0038	0.9999
	XG	−7.4699 ± 0.0209	14.2086 ± 0.0143	0.0952 ± 0.0006	0.9999
Orange juice	TS	0.1623 ± 0.0016	0.7513 ± 0.0122	0.7370 ± 0.0035	0.9996
	HDP	0.8044 ± 0.0018	3.0854 ± 0.0049	0.4425 ± 0.0159	0.9999
	XG	−7.7606 ± 0.0128	15.3304 ± 0.0479	0.0778 ± 0.0008	0.9987

* Value are mean ± standard deviation; *n* = 3. TS: tapioca starch; HDP: hydroxypropyl distarch phosphate; XG: xanthan gum.

**Table 4:** Regression models of power law and Casson models for thickened fluid matrices formulated with different thickeners and aquatic continuous phases (bases) *.

Base	Thickener	Power Law Model			Casson Model	
		*K* (Pa·s^n^)	*n*	R^2^	σ_0_ (Pa) *	R^2^
DW	TS	0.9334 ± 0.0190 ^A,a^	0.5835 ± 0.0094 ^A,a^	0.9986	0.4047 ± 0.0007 ^A,a^	0.9903
	HDP	2.4421 ± 0.0052 ^A,b^	0.4773 ± 0.0086 ^A,b^	0.9989	1.3528 ± 0.0072 ^A,b^	0.9865
	XG	7.3549 ± 0.0959 ^A,c^	0.1483 ± 0.0048 ^A,c^	0.9974	6.3973 ± 0.2226 ^A,c^	0.9139
SP	TS	0.8482 ± 0.0020 ^B,a^	0.6806 ± 0.0059 ^B,a^	0.9997	0.2538 ± 0.0008 ^B,a^	0.9948
	HDP	2.9300 ± 0.0193 ^B,b^	0.4524 ± 0.0210 ^A,b^	0.9974	1.7450 ± 0.0159^B,b^	0.9894
	XG	6.7255 ± 0.0493 ^B,c^	0.1772 ± 0.0082 ^B,c^	0.9972	5.6551 ± 0.3009 ^B,c^	0.9121
OJ	TS	0.8522 ± 0.0059 ^B,a^	0.7048 ± 0.0042 ^C,a^	0.9993	0.2438 ± 0.0041 ^C,a^	0.9958
	HDP	3.8295 ± 0.3158 ^C,b^	0.3940 ± 0.0101 ^B,b^	0.9990	2.4338 ± 0.0262 ^C,b^	0.9788
	XG	7.5538 ± 0.0760 ^C,c^	0.1432 ± 0.0074 ^A,c^	0.9970	6.6054 ± 0.0289 ^A,c^	0.9120

* Values are mean ± standard deviation; *n* = 3. The uppercase letters indicate significant difference using the same thickener (*p* < 0.05). The lowercase letters indicate significant difference using the same aquatic continuous phase (*p* < 0.05). DW: distilled water; SD: sport drink; OJ: orange juice; TS: tapioca starch; HDP: hydroxypropyl distarch phosphate; XG: xanthan gum.

**Table 5:** Linear regression outputs of equation G′ = kw*^n^* in log scale for thickened fluid with different combinations of continuous bases, thickeners (TCN), and processes with the frequency range between 0.1 and 10 Hz.

Base	TCN	Holding Time	G′			G″		
			k	*n*	R^2^	k	*n*	R^2^
DW	TS	3 min	0.2245 ± 0.0342 *	0.5345 ± 0.0333 *	0.9930 *	0.4410 ± 0.0515	0.6157 ± 0.0120	0.9939
		6 min	0.0965 ± 0.1101 *	0.8726 ± 0.3447 *	0.9984 *	0.3605 ± 0.0903	0.5668 ± 0.0108	0.9974
	HDP	3 min	1.5238 ± 0.0875	0.1980 ± 0.0291	0.9432	1.0140 ± 0.0806	0.3594 ± 0.0071	0.9905
		6 min	1.6478 ± 0.1059	0.1622 ± 0.0291	0.9022	1.0444 ± 0.1102	0.3410 ± 0.0046	0.9912
	XG	3 min	0.9825 ± 0.0669 *	0.3911 ± 0.0077 *	0.9913 *	0.6806 ± 0.0816	0.2203 ± 0.0083	0.9836
		6 min	0.9098 ± 0.1449 *	0.3909 ± 0.0116 *	0.9933 *	0.6883 ± 0.0556	0.2231 ± 0.0037	0.9795
SD	TS	3 min	−0.3227 ± 0.0527	1.6479 ± 0.1133	0.9962	0.0365 ± 0.0030	0.7167 ± 0.0468	0.9858
		6 min	−0.0539 ± 0.0250 *	0.5138 ± 0.0449 *	0.9861 *	0.2947 ± 0.0380	0.6175 ± 0.0108	0.9810
	HDP	3 min	1.3947 ± 0.0857	0.2644 ± 0.0749	0.9694	0.8313 ± 0.0864	0.3788 ± 0.0107	0.9916
		6 min	1.4134 ± 0.1548	0.2398 ± 0.0362	0.9421	0.8618 ± 0.0973	0.3778 ± 0.0361	0.9931
	XG	3 min	1.1480 ± 0.0559	0.2777 ± 0.0682	0.9482	1.1741 ± 0.0430	0.2539 ± 0.0839	0.9670
		6 min	0.5922 ± 0.0431	0.1294 ± 0.0169	0.9832	0.6130 ± 0.0440	0.1338 ± 0.0227	0.9417
OJ	TS	3 min	−0.3256 ± 0.0174	1.7310 ± 0.0392	0.9986	0.0066 ± 0.0287	0.6608 ± 0.0145	0.9995
		6 min	−0.3566 ± 0.0374	1.7084 ± 0.0623	0.9982	−0.0056 ± 0.0049	0.6852 ± 0.0296	0.9969
	HDP	3 min	0.8121 ± 0.0153	0.7539 ± 0.0000	0.9105	0.5112 ± 0.0153	0.4943 ± 0.0000	0.9959
		6 min	0.8966 ± 0.1121	0.5778 ± 0.1761	0.9077	0.5394 ± 0.0775	0.3310 ± 0.1414	0.9960
	XG	3 min	1.0803 ± 0.0554	0.3471 ± 0.0696	0.9928	0.5680 ± 0.0671	0.1541 ± 0.0084	0.9822
		6 min	0.8686 ± 0.0400	0.5744 ± 0.0899	0.9009	0.4370 ± 0.0093	0.2724 ± 0.1110	0.9793

DW: distill water; SD: sport drink; OJ: orange juice; TS: tapioca starch; HDP: hydroxypropyl distarch phosphate; XG: xanthan gum. Value are mean ± standard deviation; *n* = 3. * indicates that the range is 0.1 to 1 Hz.

**Table 6:** Storage modulus (G′), loss modulus (G″), and loss tangent (*tanδ*) affected by different combinations of holding times (T in minutes), commercial thickeners, and aquatic continuous phases at 1 Hz.

Base	T	TS			HDP			XG		
		G′ (Pa)	G″ (Pa)	*tanδ*	G′ (Pa)	G″ (Pa)	*tanδ*	G′ (Pa)	G″ (Pa)	*tanδ*
DW	3	1.62 ± 0.116 ^A,a^	2.87 ± 0.342 ^A,a^	1.75 ± 0.105 ^A,a^	34.07 ± 6.890 ^B,a^	10.24 ± 1.820 ^B,a^	0.31 ± 0.105 ^B,a^	9.60 ± 1.328 ^A,bc^	5.02 ± 0.859 ^A,a^	0.52 ± 0.022 ^B,a^
	6	1.50 ± 0.058 ^A,a^	2.36 ± 0.418 ^A,ab^	1.56 ± 0.230 ^A,b^	46.36 ± 8.144 ^B,a^	11.37 ± 2.89 ^B,a^	0.25 ± 0.003 ^B,a^	9.52 ± 1.143 ^A,bc^	5.00 ± 0.629 ^A,a^	0.52 ± 0.005 ^B,a^
SD	3	0.86 ± 0.056 ^A,b^	1.18 ± 0.044 ^A,c^	1.39 ± 0.140 ^A,a^	24.98 ± 6.010 ^B,ab^	6.98 ± 1.54 ^B,ab^	0.28 ± 0.005 ^C,a^	14.10 ± 1.826 ^AB,ab^	3.85 ± 0.393 ^A,ab^	0.28 ± 0.011 ^C,b^
	6	0.84 ± 0.071 ^A,b^	1.97 ± 0.183 ^A,b^	2.40 ± 0.386 ^B,a^	27.59 ± 8.528 ^B,ab^	7.38 ± 1.59 ^B,ab^	0.29 ± 0.044 ^C,a^	13.91 ± 2.940 ^AB,a^	4.02 ± 0.425 ^A,ab^	0.27 ± 0.002 ^C,b^
OJ	3	0.8 2± 0.058 ^A,b^	1.03 ± 0.069 ^A,c^	1.26 ± 0.038 ^A,a^	7.51 ± 0.263 ^B,b^	3.16 ± 0.111 ^B,b^	0.42 ± 0.000 ^B,b^	12.13 ± 1.807 ^B,abc^	3.68 ± 0.575 ^B,ab^	0.30 ± 0.003 ^B,b^
	6	0.80 ± 0.056 ^A,b^	1.03 ± 0.005 ^A,c^	1.30 ± 0.103 ^A,a^	8.96 ± 1.922 ^B,b^	3.50 ± 0.677 ^B,b^	0.39 ± 0.014 ^B,b^	7.75 ± 0.376 ^B,c^	2.66 ± 0.058 ^B,b^	0.35 ± 0.019 ^B,c^

DW: distill water; SD: sport drink; OJ: orange juice; TS: tapioca starch; HDP: hydroxypropyl distarch phosphate; XG: xanthan gum. The uppercase letters indicate significant difference using the same base (*p* < 0.05). The lowercase letters indicate significant difference using the same thickener (*p* < 0.05). Value are mean ± standard deviation; *n* = 3.

**Table 7:** Textural parameter variations due to thermal processing, aquatic continuous phases, and commercial thickeners after 28 days of storage at 4 °C facilitated by a 121 °C-15 min thermal process.

Parameter			Apparent Viscosity	Viscosity Index	Consistency Index
Process	Formula		(mPa.s)	(g.s)	(g.s)
	Base	Thickener			
Control	DW	TS	315.5 ± 5.70 ^A,a^	177.5 ± 3.52 ^A,a^	266.1 ± 10.62 ^A,a^
		HDP	316.9 ± 9.33 ^AB,a^	279.4 ± 1.00 ^A,b^	427.5 ± 8.21 ^A,b^
		XG	307.1 ± 9.36 ^A,a^	168.8 ± 16.76 ^A,c^	386.3 ± 9.32 ^A,c^
	SD	TS	303.7 ± 3.12 ^B,a^	167.7 ± 2.48 ^B,a^	274.8 ± 9.87 ^AB,a^
		HDP	325.9 ± 4.65^A,b^	287.4 ± 10.09 ^B,b^	319.1 ± 16.88 ^B,b^
		XG	308.8 ± 6.87 ^A,a^	286.2 ± 6.19 ^B,b^	562.3 ± 9.59 ^B,c^
	OJ	TS	305.0 ± 5.36 ^B,a^	109.1 ± 9.84 ^A,a^	292.4 ± 13.05 ^B,a^
		HDP	309.8 ± 5.55 ^B,a^	182.5 ± 5.01 ^C,b^	364.8 ± 5.34 ^C,b^
		XG	306.1 ± 6.11 ^A,c^	290.9 ± 7.44 ^C,c^	497.7 ± 12.98 ^C,c^
121 °C	DW	TS	44.2 ± 4.50 ^A,a,^**	43.4 ± 1.76 ^A,a,^**	266.5 ± 3.53 ^A,a^
15 min		HDP	227.8 ± 4.61 ^A,b,^**	65.5 ± 17.00 ^A,b,^**	266.1 ± 1.00 ^A,c,^**
		XG	107.1 ± 1.70 ^A,c,^**	86.2 ± 2.09 ^A,c,^**	252.7 ± 16.76 ^A,b,^**
	SD	TS	4.53 ± 0.98 ^B,a,^**	30.33 ± 2.64 ^B,a,^**	232.0 ± 3.53 ^B,a,^**
		HDP	9.1 ± 1.93 ^B,b,^**	23.7 ± 2.26 ^B,a,^**	241.6 ± 2.59 ^B,a,^*
		XG	36.8 ± 2.60 ^B,c,^**	68.9 ± 5.60 ^B,b,^**	260 ± 10.31 ^A,b,^**
	OJ	TS	5.7 ± 1.61 ^B,a,^**	53.9 ± 2.26 ^C,a,^**	350.8 ± 9.19 ^C,a,^**
		HDP	6.3 ± 0.98 ^B,a,^**	50.64 ± 3.68 ^A,a,^**	345.7 ± 4.83 ^C,a,^**
		XG	27.8 ± 2.60 ^C,b,^**	63.4 ± 5.24 ^B,b,^**	266.2 ± 12.85 ^A,b,^**

DW: distill water; SD: sport drink; OJ: orange juice; TS: tapioca starch; HDP: hydroxypropyl distarch phosphate; XG: xanthan gum. The superscript letters in uppercase indicate the significant differences observed as the same thickener is incorporated with the same thermal process. The superscript letters in lowercase indicate the significant differences observed in the same aquatic base group with the same thermal process. * and ** indicate significant differences at 95% and 99% confidence levels, respectively, comparing to the corresponding control data.

**Table 8:** Textural parameter variations due to thermal processing, aquatic continuous phases, and commercial thickeners prior to and after a 28 day storage at 4 °C facilitated by thermal processes of 80 °C-3/6 min.

Parameter			Apparent Viscosity		Viscosity Index		Consistency Index	
Process	Formula		(mPa.s)		(g.s)		(g.s)	
	Base	Thickener	Day 0	Day 28	Day 0	Day 28	Day 0	Day 28
80 °C	DW	TS	239.3 ± 5.20 ^A,a,^**	226.9 ± 11.49	116.6 ± 2.26 ^A,a^	80.2 ± 3.04 ^¶^	255.3 ± 10.15 ^A,a^	280.4 ± 0.17 ^¶^
3 min		HDP	285.6 ± 9.17 ^BA,b,^*	339.6 ± 10.42 ^¶^	275.3 ± 11.06 ^A,b^	281.1 ± 3.20	422.5 ± 2.93 ^A,b^	446.3 ± 20.68
		XG	181.3 ± 3.93 ^A,c,^**	189.4 ± 6.05	178.23 ± 16.59 ^A,c^	194.4 ± 4.64	391.5 ± 3.43 ^A,c^	451.51 ± 5.99 ^¶^
	SD	TS	162.6 ± 5.97 ^B,a,^**	121.5 ± 4.15 ^¶^	95.7 ± 0.84 ^B,a,^**	92.0 ± 1.89	287.8 ± 11.97 ^B,a^	292.5 ± 14.13
		HDP	378.5 ± 0.98 ^B,b,^**	331.0 ± 15.29 ^¶^	171.1 ± 6.17 ^B,b,^**	199.1 ± 5.64 ^¶^	326.3 ± 5.28 ^B,b^	380.9 ± 5.99 ^¶^
		XG	300.3 ± 2.60 ^B,c^	250.3 ± 11.91 ^¶^	284.4 ± 4.47 ^B,c^	291.3 ± 9.17	572.1 ± 12.31 ^B,c^	574.5 ± 12.31
	OJ	TS	174.8 ± 5.31 ^C,a,^**	185.3 ± 13.60 ^¶^	103.1 ± 1.10 ^C,a^	86.6 ± 1.34 ^¶^	295.5 ± 5.38 ^B,a^	290.6 ± 12.36
		HDP	330.7 ± 4.25 ^C,b,^**	416.1 ± 15.28 ^¶^	129.8 ± 1.33 ^C,b^	122.0 ± 6.77	370.6 ± 8.40 ^C,b^	330.1 ± 23.58 ^¶^
		XG	261.8 ± 5.10 ^C,c,^**	273.7 ± 2.49	283.6 ± 5.38 ^B,c^	320.1 ± 7.81 ^¶^	484.6 ± 9.91 ^C,c^	540.0 ± 7.65 ^¶^
80 °C	DW	TS	270.1 ± 21.79 ^A,a,^*	211.8 ± 7.20 ^¶^	114.3 ± 2.35 ^A,a^	91.2 ± 1.60 ^¶^	260.0 ± 2.79 ^A,a^	283.5 ± 4.05 ^¶^
6 min		HDP	340.1 ± 16.81 ^A,b^	406.8 ± 11.68 ^¶^	280.0 ± 4.26 ^A,b^	337.3 ± 3.02 ^¶^	428.04 ± 9.20 ^A,b^	475.8 ± 7.42 ^¶^
		XG	174.7 ± 2.67 ^A,c,^**	181.5 ± 2.61 ^¶^	179.8 ± 0.76 ^A,c^	167.9 ± 9.32	389.4 ± 5.02 ^A,c^	388.8 ± 34.97
	SD	TS	118.4 ± 1.96 ^B,a,^**	81.7 ± 3.76 ^¶^	96.6 ± 0.99 ^B,a,^**	85.3 ± 3.00 ^¶^	278.9 ± 16.35 ^AB,a^	283.6 ± 0.55
		HDP	366.8 ± 19.79 ^A,b^	374.8 ± 7.20	169.4 ± 4.17 ^B,b,^**	192.0 ± 2.95 ^¶^	340.8 ± 6.11 ^B,b^	400.9 ± 13.99 ^¶^
		XG	292.4 ± 1.70 ^B,c,^*	249.3 ± 12.44 ^¶^	261.3 ± 2.54 ^B,c,^*	286.5 ± 6.52 ^¶^	566.2 ± 9.79 ^B,c^	596.6 ± 12.91
	OJ	TS	148.5 ± 7.67 ^C,a,^**	117.5 ± 1.33 ^¶^	100.4 ± 2.51 ^AB,a^	101.9 ± 2.93	286.8 ± 3.28 ^B,b^	292.6 ± 2.72
		HDP	288.4 ± 5.46 ^B,b,^**	284.6 ± 4.77	187.1 ± 7.50 ^C,b^	199.9 ± 3.61 ^¶^	352.9 ± 8.89 ^B,c^	412.7 ± 3.46 ^¶^
		XG	286.2 ± 9.36 ^B,b,^*	277.1 ± 5.57 ^¶^	305.5 ± 1.38 ^C,c^	333.8 ± 9.20 ^¶^	525.1 ± 9.35 ^B,c^	532.2 ± 6.06

DW: distilled water; SD: sport drink; OJ: orange juice; TS: tapioca starch; HDP: hydroxypropyl distarch phosphate; XG: xanthan gum. The superscript letters in uppercase indicate the significant differences observed as the same thickener is incorporated with the same thermal process. The superscript letters in lowercase indicate the significant differences observed in the same aquatic base group with the same thermal process. * and ** indicate significant differences at 95% and 99% confidence levels, respectively, comparing to the corresponding control data. ^¶^ indicates significant difference at the end of a 28 day storage comparing to day 0.

## Data Availability

Data are contained within the article or supplemented upon request from the section editors.
